# Supplementary material for: Structural convergence and water-mediated substrate mimicry enable broad neuraminidase inhibition by human antibodies
Source: Nat Commun. 2025 Aug 1;16:7068. doi: 10.1038/s41467-025-62339-z (PMC12316982; doi:10.1038/s41467-025-62339-z)
Supplement: Supplementary file 1 — Supplementary Information [file 41467_2025_62339_MOESM1_ESM.pdf]

Supplementary Information for

Structural Convergence and Water-Mediated Substrate Mimicry Enable Broad Neuraminidase Inhibition by Human Antibodies

Julia Lederhofer,[1,†] Andrew J. Borst,[2,3,†] Lam Nguyen,[1] Rebecca A. Gillespie,[1] Connor J. Williams,[4] Emma L. Walker,[4] Julie E. Raab,[1] Christina Yap,[1] Daniel Ellis,[2,3,5] Adrian Creanga,[1] Hyon-Xhi Tan,[6] Thi H. T. Do,[6] Michelle Ravichandran,[1] Adrian B. McDermott,[1] Valerie Le Sage,[4] Sarah F. Andrews,[1] Barney S. Graham,[1] Adam K. Wheatley,[6] Douglas S. Reed,[4] Neil P. King,[2,3,*] and Masaru Kanekiyo[1,*]

[1]Vaccine Research Center, National Institute of Allergy and Infectious Diseases, National Institutes of Health, Bethesda, MD 20892, United States.
[2]Institute for Protein Design, University of Washington, Seattle, WA 98195, United States.
[3]Department of Biochemistry, University of Washington, Seattle, WA 98195, United States.
[4]Department of Immunology, Center for Vaccine Research, University of Pittsburgh, Pittsburgh, PA, USA.
[5]Graduate Program in Molecular and Cellular Biology, University of Washington, Seattle, WA 98195, United States.
[6]Department of Microbiology and Immunology, Peter Doherty Institute for Infection and Immunity, University of Melbourne, Melbourne, VIC 3000, Australia.

*Correspondence to: neilking@uw.edu (N.P.K.), kanekiyom@nih.gov (M.K.).

[†]These authors contributed equally to this work.

This file contains:

Supplementary figures 1–6
Supplementary tables 1–2
Supplementary reference 1

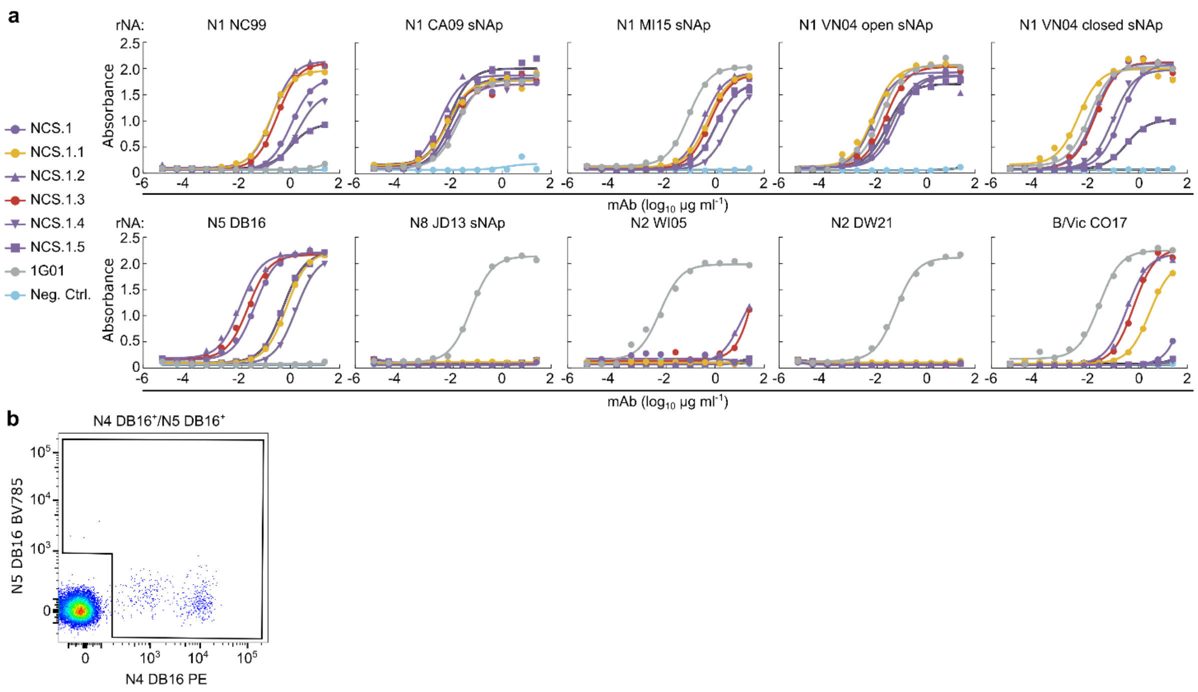

Supplementary fig. 1. ELISA binding curves and flow cytometry analysis of NA-specific memory B cells from donor A.

a ELISA binding curves of NCS.1.x mAbs to recombinant NAs from group 1, group 2, and IBV strains. Negative control: D25 (anti-respiratory syncytial virus site Ø mAb). The experiment was repeated at least once with similar results, and representative results are shown. **b** Flow cytometry plots showing NA-specific IgG+ memory B cells from donor A, sorted with N4 DB16 and N5 DB16 probes. Flow cytometry gating strategy was originally published in Casalino et al.[1]

a

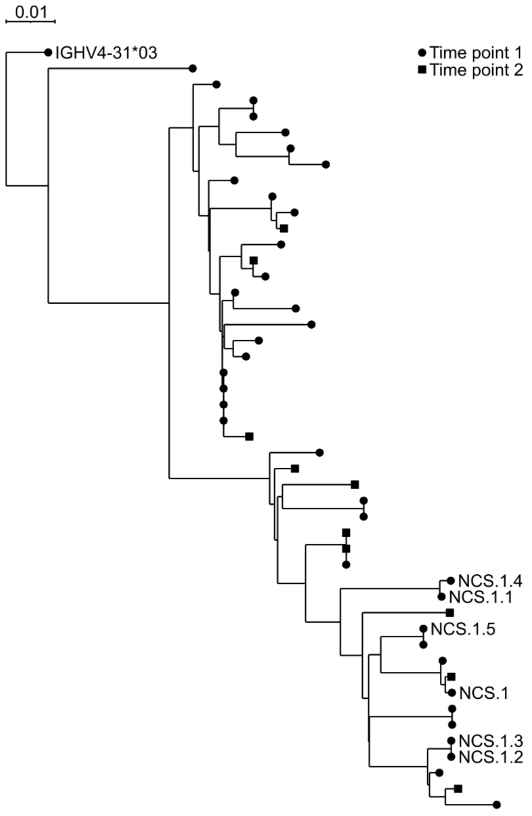

b

```
VH4-31*01   caggtgcagctgcaggagtcgggcccaggactggtgaagccttcacagaccctgtccctc 60
NCS.1 HC    caggtgcagttgccggagtcgggcccacgactggtgaagccttcagagaccctgtccctc 60
NCS.1.1 HC  caggtgcagctgcaggagtcgggcccacggctggtgaagccttcagagaccctgtccctc 60
NCS.1.3 HC  caggtgcagctacaggagtcgggcccacgactggtgaagccttcagagaccctgtccctc 60
            ********* * * ************* * *************** **************

VH4-31*01   acctgcactgtctctggtggctccatcagcagtggtggttactactggagctggatccgc 120
NCS.1 HC    acgtgcagtgtctctggtgagtccattagtagtggtggttactactggacctggatccgc 120
NCS.1.1 HC  acgtgcagtgtctctggtgagtccatcagcagtggtggttactactggacctggatccgc 120
NCS.1.3 HC  acgtgcagtgtctctggtgagtccattagcagtggtggttattactggacctggatccgc 120
            ** **** ***********  ***** ** *********** ******* **********

VH4-31*01   cagcacccagggaagggcctggagtggattgggtacatctattacagtgggagcacctac 180
NCS.1 HC    cagcacccagggaagggcctggagtggattgggaacatctttgacactgggagcacccac 180
NCS.1.1 HC  cagcacccagggaagggcctggagtggattgggaacatctttgacactgggagcaccgac 180
NCS.1.3 HC  cagcacccagggaagggcctggagtggattgggaacatctttgacactgggagcaccgac 180
            ********************************* ****** * *** ********** **

VH4-31*01   tacaacccgtccctcaagagtcgagttaccatatcagtagacacgtctaagaaccagttc 240
NCS.1 HC    tacagtccgtccctcaagacgcgactgacgatatcgatagatacgtctaagaaccagttc 240
NCS.1.1 HC  tacagtccgtccctcaagacgcgacttacgatatcgatagacacgtctaagaaccagttc 240
NCS.1.3 HC  tacagtccgtccctcaagacacgactgacgatatcgatagacacgtctaagaaccagttc 240
            ****  *************  *** * ** *****  **** ******************

VH4-31*01   ccct-gaagctgagctctgtgactgccgcggacacggccgtgtattactgtgcgagaga  298
NCS.1 HC    tacctgaggctgaactctgccacggccgcggacacggccgtgtattattgtgcgagagt  299
NCS.1.1 HC  tacttgcggctgaactctgcgacggccgcggacacggccgtgtattattgtgcgagagt  299
NCS.1.3 HC  tacctgaggttgacctctgcgacggccgcggacacggccgtttattattgtgcgagagt  299
              *  *  * *** *****  ** ***************** ***** **********


IGKV2-28*01 gatattgtgatgactcagtctccactctccctgcccgtcacccctggagagccggcctcc 60
NCS.1 KC    gatattgtgatgactcagtctccatcctccctgcccgtcacccctggagagccggcctcc 60
NCS.1.1 KC  gatattgtgatgactcagtctccattctccctgcccgtcacccctggagagccggcctcc 60
NCS.1.3 KC  gatattgtgatgactcagtctccatttaccctgcccgtcacccctggagagccggcctcc 60
            ************************    ********************************

IGKV2-28*01 atctcctgcaggtctagtcagagcctcctgcatagtaatggatacaactatttggattgg 120
NCS.1 KC    atctcctgcaggtctagtcagagcctcctgcatagtaatggatacacctatttggattgg 120
NCS.1.1 KC  atctcttgcaggtctagtcagagcctcctgcatagtaatggatacacctatttggattgg 120
NCS.1.3 KC  atctcctgcaggtctagtcagagcctcctgcatagtaatggatacacctatttggattgg 120
            ***** **************************************** *************

IGKV2-28*01 tacctgcagaagccagggcagtctccacagctcctgatctatttgggttctaatcgggcc 180
NCS.1 KC    tacctgcagaagccagggcagtctccacaactcctgatctatttggcttcgaatcgggcc 180
NCS.1.1 KC  tacctgcagaagccagggcagtctccacaactcctgatctctttcacttcgaatcgggcc 180
NCS.1.3 KC  tacctgcagaagccagggcagtctccacaactcctgatctattttgcttcgaatcgggcc 180
            ***************************** ********** ***   *** *********

IGKV2-28*01 tccggggtccctgacaggttcagtggcagtggatcaggcacagattttacactgaaaatc 240
NCS.1 KC    tccggggtccctgacaggttcagtggcagtggatccggcacatattttacactgaaaatc 240
NCS.1.1 KC  tccggggtccctgacaggttcagtggcagtggctccggcacatattttacactgaaaatc 240
NCS.1.3 KC  tccggggtccctgacaggttcagtggcagtggatccggcacatattttacactgaaaatc 240
            ******************************** ** ****** *****************

IGKV2-28*01 agcagagtggaggctgaggatgttggggtttattactgcatgcaagctctacaaactcct 300
NCS.1 KC    agtagagtggaggctgaagatgttggggtttactactgcatgcaagctgtccaaactccg 300
NCS.1.1 KC  agtagagtggaggctgaagatgttggggtttactactgcatgcaagctgtccaaactccg 300
NCS.1.3 KC  agtagagtggaggctgaagatgttggggtttactactgcatgcaagctgtccaatctccg 300
            ** ************** ************** *************** * *** ****
```

Supplementary fig. 2. Phylogenetic tree analysis and nucleotide sequence alignment of NCS.1.x.

a Phylogenetic tree analysis of NCS.1.x antibody heavy chain gene. **b** Nucleotide sequence alignment of NCS.1, NCS.1.1, and NCS.1.3 heavy and light chains.

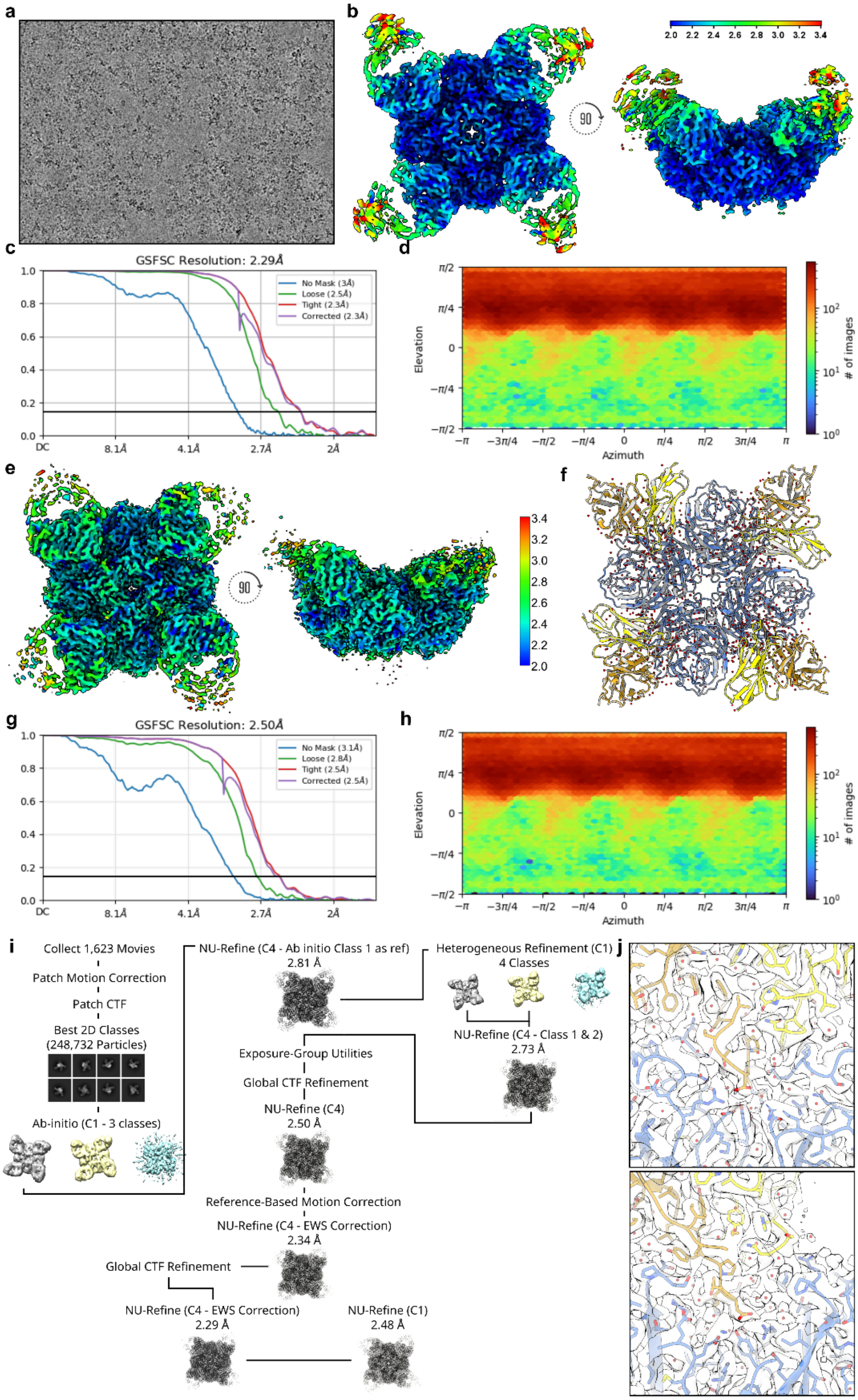

a
b
90
2.0 2.2 2.4 2.6 2.8 3.0 3.2 3.4
c
GSFSC Resolution: 2.29Å
No Mask (3Å)
Loose (2.5Å)
Tight (2.3Å)
Corrected (2.3Å)
DC
8.1Å
4.1Å
2.7Å
2Å
d
Elevation
Azimuth
# of images
e
90
f
g
GSFSC Resolution: 2.50Å
No Mask (3.1Å)
Loose (2.8Å)
Tight (2.5Å)
Corrected (2.5Å)
h
Elevation
Azimuth
# of images
i
Collect 1,623 Movies
Patch Motion Correction
Patch CTF
Best 2D Classes
(248,732 Particles)
Ab-initio (C1 - 3 classes)
NU-Refine (C4 - Ab initio Class 1 as ref)
2.81 Å
Heterogeneous Refinement (C1)
4 Classes
NU-Refine (C4 - Class 1 & 2)
2.73 Å
Exposure-Group Utilities
Global CTF Refinement
NU-Refine (C4)
2.50 Å
Reference-Based Motion Correction
NU-Refine (C4 - EWS Correction)
2.34 Å
Global CTF Refinement
NU-Refine (C4 - EWS Correction)
2.29 Å
NU-Refine (C1)
2.48 Å
j

Supplementary fig. 3. NCS.1.1/N1-CA09-sNAp cryoEM data processing statistics.
a Representative raw micrograph shown from 2,212 total collected movies of the NCS.1.1/N1 complex. C4 reconstruction showing ideal particle distribution and contrast. **b** CryoEM local resolution map from the C4 reconstruction calculated using an FSC value of 0.143, viewed along two different angles. Local resolution estimates range from ~2.0 Å at the core of N1 to ~2.5 Å along the periphery of the NCS.1.1 Fv. The binding interface of NCS.1.1 and N1-CA09-sNAp was resolved to a nominal resolution of ~2.0 Å. **c** Global resolution estimation plot for the C4 reconstruction. **d** Orientational distribution plot for the C4 reconstruction. **e** Local resolution map from the C1 reconstruction, viewed along two orthogonal axes. Local resolution estimates range from ~2.2 Å at the core of N1 and at the NCS.1.1–N1-CA09-sNAp binding interface, out to ~2.8 Å along the periphery of the Fab scFv. **f** Built C1 structure of the NCS.1.1/N1 complex (colored) compared to the C4 reconstruction structure (grey; RMSD = 0.1 Å). Verified water molecules (as described in the methods) of the C1 reconstruction are colored in red. **g** Global resolution estimation plot for the C1 reconstruction. **h** Orientational distribution plot for the C1 reconstruction. **i** Data processing pipeline. **j** Representative map-to-model fit of the C1 reconstruction of the NCS.1.1–N1 CA09 sNAp complex, shown from two angles. The maps reveal excellent agreement at the antibody–antigen catalytic pocket interface, with several well-resolved water molecules bridging contacts across the interface.

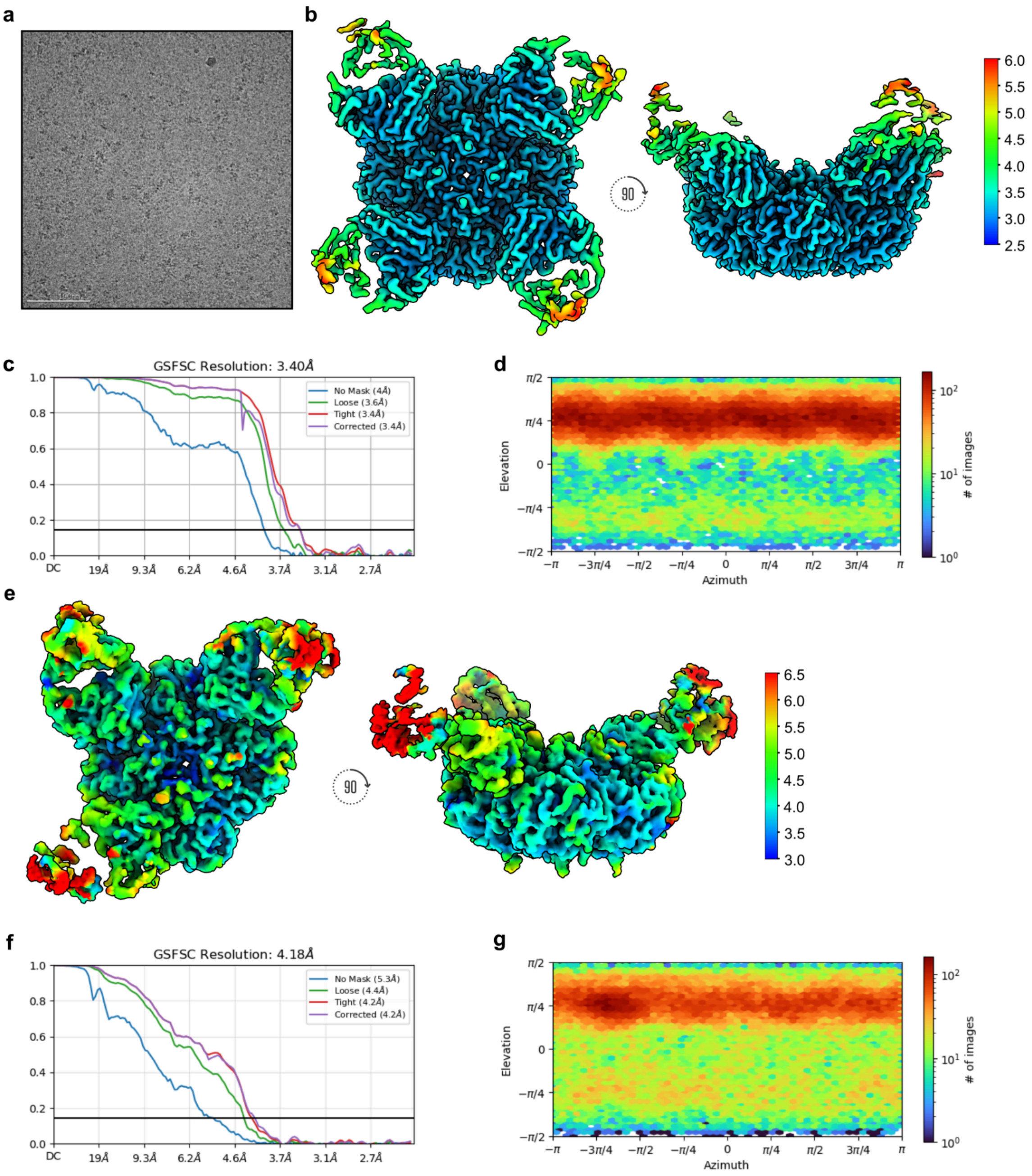

Supplementary fig. 4. CryoEM data processing statistics for NCS.1/DB16 N5 NA with four and three Fabs bound.

a Representative raw micrograph shown from 1,623 collected movies showing ideal particle distribution and contrast for the dataset used to reconstruct both NCS.1/N5 complexes. **b** CryoEM local resolution map of the N5 bound to four Fabs, calculated using an FSC value of 0.143, viewed along two different angles. Local resolution estimates range from ~2.5 Å at the core of N5 to ~3.5 Å along the periphery of the NCS.1 Fv. The binding interface of NCS.1 and N5 was resolved to a nominal resolution of ~3.0 Å. **c** Global resolution estimation plot of N5 bound to four Fabs. **d** Orientational distribution plot of N5 bound to four Fabs. **e** CryoEM local resolution maps of N5 bound to three Fabs, calculated using an FSC value of 0.143, viewed along two different angles. Local resolution estimates range from ~3.5 Å at the core of N5 to ~5.0 Å along the periphery of the NCS.1 Fv. The

binding interface of NCS.1 and N5 was resolved to a nominal resolution of ~4.0 Å. **f** Global resolution estimation plots of the N5 bound to three Fabs. **g** Orientational distribution plot of N5 bound to three Fabs.

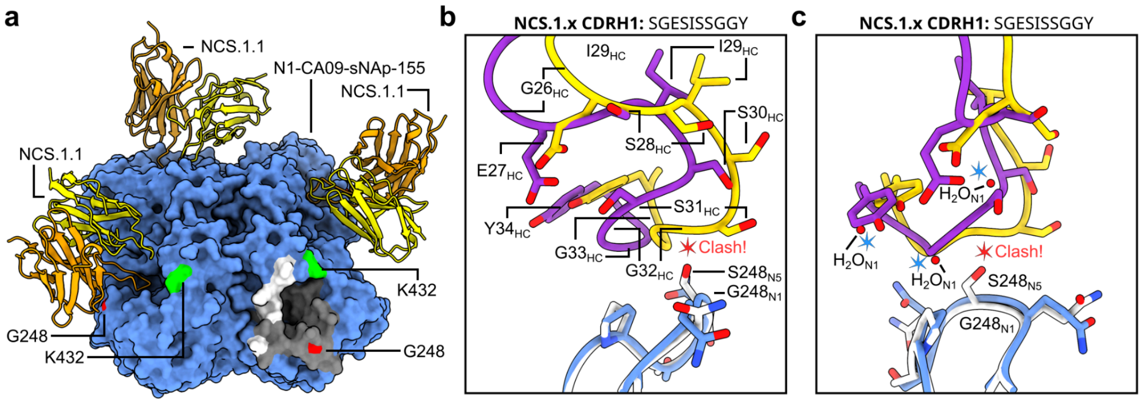

Supplementary fig. 5. Structural basis for accommodation of G248-escape mutations and CDRH1 flexibility in NCS.1.x mAbs.

a Epitope map of the NCS.1.1 mAb, rendered similarly to Fig. 2e. The heavy chain footprint is shown in grey, and additional light-chain–only contacts are indicated in white. The location of K432, a common site of escape mutation that lies distal to the mAb binding interface, is highlighted in green. In contrast, G248, which is centrally located within the heavy chain footprint, is highlighted in red. **b** Top: Amino acid sequence of the CDRH1 loop. Bottom: Structural overlay of the CDRH1 loops from NCS.1.x mAbs bound to NA with either G248 (present in the N1 structure) or the G248S mutant (present in the N5 structure). The CDRH1 loop from the N1 structure is shown in gold, and the CDRH1 loop from the N5 structure is shown in purple. The serine substitution introduces a predicted steric clash (denoted in red) with the CDRH1 conformation observed in the N1 complex. The glycine-serine–rich nature of the CDRH1 motif enables conformational adaptation, as seen in the N5 structure, allowing the loop to accommodate bulkier side chains at position 248. **c** Top: Amino acid sequence of the CDRH1 loop. Bottom: Overlay of NCS.1.x structures illustrating how CDRH1 rearrangement in response to the G248S mutation displaces water molecules (denoted in blue) previously observed in the N1 complex. This displacement preserves interactions that would otherwise be solvent-mediated.

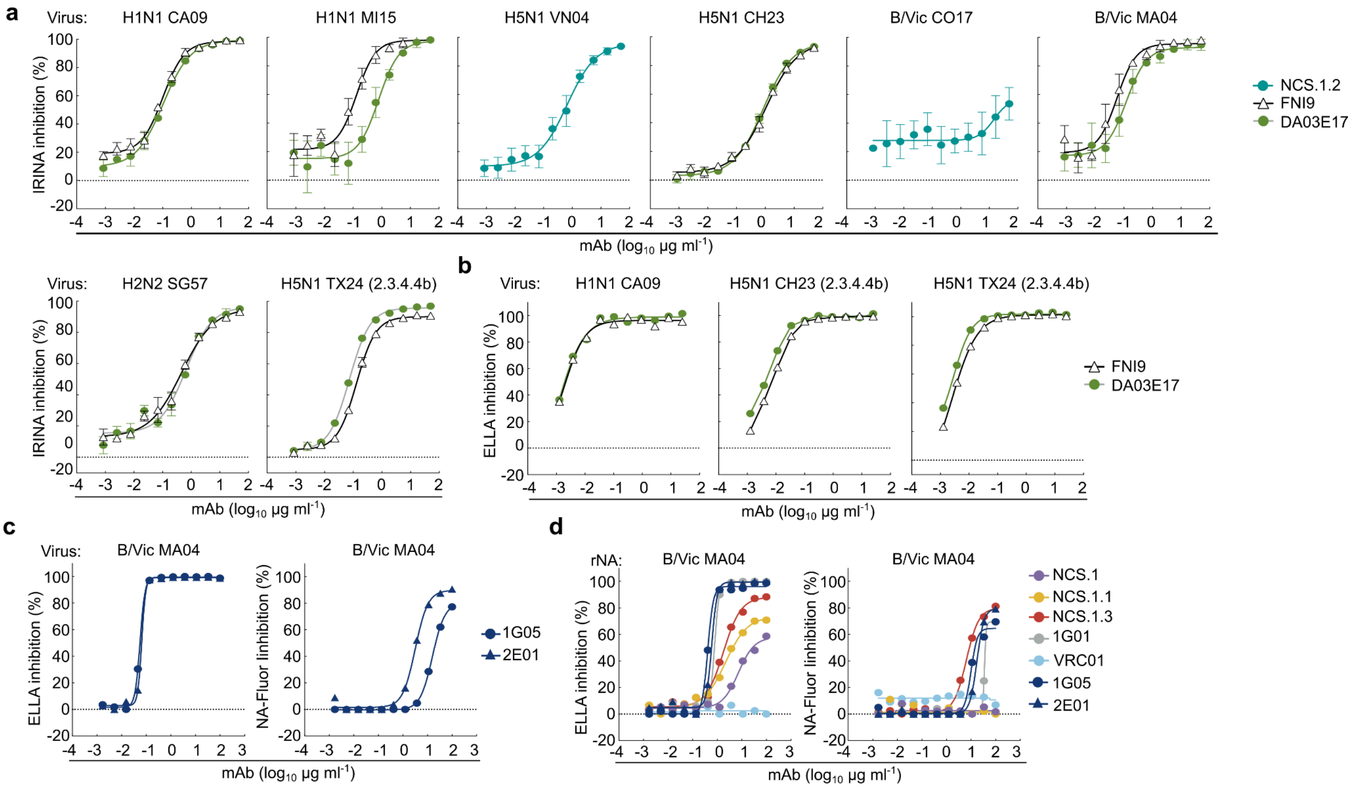

Supplementary fig. 6. NAI activity of NCS.1.x and known NA catalytic site mAbs across various assays and viral strains.

a NAI activity of the mAbs NCS.1.2, FNI9, and DA03E17 measured by IRINA. Reporter viruses used include H1N1 CA09, H1N1 MI15, H5N1 VN04, H5N1 CH23, B/Vic CO17, B/Vic MA04, H2N2 SG57, and H5N1 TX24. Data are plotted as mean ± SD of n = 4 individual wells at each dilution. **b** NAI activity of the mAbs FNI9 and DA03E17 measured by ELLA. Reporter viruses used include H1N1 CA09, H5N1 CH23, and H5N1 TX24. Data are plotted as the mean of n = 2 individual wells at each dilution. **c** NAI activity of control mAbs 1G05 and 2E01 measured by ELLA and NA-Fluor assay with B/Vic MA04 virus. Data are plotted for each dilution. **d** NAI activity of the mAbs measured by ELLA and NA-Fluor assay with recombinant B/Vic MA04 neuraminidase. Data are plotted for each dilution. All experiments were performed once including appropriate controls. Source data are provided as a Source Data file.

Supplementary table 1. CryoEM data collection, refinement and validation statistics

	NCS.1.1/N1 (4 fabs - C4) PDB: 9EJF EMD-48102	NCS.1.1/N1 (4 fabs - C1) PDB: 9O9V EMD-70264	NCS.1/N5 (4 fabs - C4) PDB:9EIT EMDB-48093	NCS.1/N5 (3 fabs - C1) PDB:9EJE EMDB-48101
**Data collection and processing**				
Magnification	105,000 ×	105,000 ×	36,000 ×	36,000 ×
Voltage (kV)	300	300	200	200
Electron exposure (e–/Å^2)	50	50	60	60
Defocus range (μm)	0.8–1.8	0.8–1.8	0.8–1.8	0.8–1.8
Pixel size (Å)	0.843	0.843	1.16	1.6
Symmetry imposed	C4	C1	C4	C1
Initial particle images (no.)	648,236	648,236	286,775	286,775
Final particle images (no.)	382,836	382,836	93,151	75,643
Map resolution (Å)	2.29	2.5	3.36	4.18
FSC threshold	0.143	0.143	0.143	0.143
Map resolution range (Å)	2.0–2.5	2.1–3.0	2.5–3.5	3.5–5.0
**Refinement**				
Initial model used (PDB code)	N/A	N/A	6Q23	6Q23
Model resolution (Å)	2.29	2.5	3.36	4.18
FSC threshold	0.143	0.143	0.143	0.143
Model resolution range (Å)	2.0–2.5	2.1–3.0	2.5–3.5	3.5–5.0
Map sharpening *B* factor (Å^2)	84.9	79.3	144.9	129.4
Model composition				
Non-hydrogen atoms	20,358	20,358	19,512	17,649
Protein residues	2,484	2,484	2,488	2,257
Ligands	BMA:4 NAG:16 CA:8	BMA:4 NAG:16 CA:8	BMA: 0 NAG: 8 CA: 4	BMA: 4 NAG: 12 CA: 4
B factors (Å^2)				
Protein	98.57	98.57	97.63	97.07
Ligand	53.04	53.04	50	50
R.m.s. deviations				
Bond lengths (Å)	0.008	0.007	0.008	0.004
Bond angles (°)	1.141	1.228	1.252	0.650
Validation				
MolProbity score	1.81	2.05	1.71	1.73
Clashscore	2.92	3.92	1.55	3.01
Poor rotamers (%)	2.94	0.69	2.86	3.01
Ramachandran plot				
Favored (%)	94.59	94.39	92.49	96.02
Allowed (%)	4.96	4.92	7.18	3.98
Disallowed (%)	0.45	0.69	0.32	0

Supplementary table 2. Statistical analysis of Kaplan-Meier curve comparisons

Comparator:	NCS.1	NCS.1.1	NCS.1.3	1G01	Pos ctrl
H1N1 CA09					
NCS.1	-				
NCS.1.1	NS	-			
NCS.1.3	NS	NS	-		
1G01	NS	NS	NS	-	
Pos ctrl	NS	NS	NS	NS	-
Neg ctrl	P < 0.0001*	P < 0.0001	P < 0.0001	P < 0.0001	P < 0.0001
B/Vic MA04					
NCS.1	-				
NCS.1.1	NS	-			
NCS.1.3	NS	NS	-		
1G01	NS	NS	NS	-	
Pos ctrl	NS	NS	NS	NS	-
Neg ctrl	P = 0.0006	P < 0.0001	P < 0.0001	P < 0.0001	P < 0.0001
H5N1 VN04					
NCS.1	-				
NCS.1.1	NS	-			
NCS.1.3	NS	NS	-		
1G01	NS	NS	NS	-	
Pos ctrl	P = 0.0360	NS	NS	NS	-
Neg ctrl	P < 0.0001	P < 0.0001	P < 0.0001	P < 0.0001	P = 0.0002
H5N1 TX24					
NCS.1	-				
NCS.1.1	-	-			
NCS.1.3	-	NS	-		
1G01	-	NS	NS	-	
Pos ctrl	-	P = 0.0014	P = 0.0053	P = 0.0003	-
Neg ctrl	-	P < 0.0001	P < 0.0001	P < 0.0001	P < 0.0001

*P < 0.033 is considered statistically significant by the Mantel-Cox test after Bonferroni correction.

Supplementary References

1. Casalino, L. *et al.* Breathing and tilting: Mesoscale simulations illuminate influenza glycoprotein vulnerabilities. *ACS Cent. Sci.* **8**, 1646–1663 (2022).
