# Supplementary material for: Structural convergence and water-mediated substrate mimicry enable broad neuraminidase inhibition by human antibodies
Source: Nat Commun. 2025 Aug 1;16:7068. doi: 10.1038/s41467-025-62339-z (PMC12316982; doi:10.1038/s41467-025-62339-z)
Supplement: Supplementary file 2 — Reporting Summary [file 41467_2025_62339_MOESM2_ESM.pdf]

nature portfolio

Corresponding author(s): Neil King and Masaru Kanekiyo

Last updated by author(s): Jul 11, 2025

Reporting Summary

Nature Portfolio wishes to improve the reproducibility of the work that we publish. This form provides structure for consistency and transparency in reporting. For further information on Nature Portfolio policies, see our Editorial Policies and the Editorial Policy Checklist.

Statistics

For all statistical analyses, confirm that the following items are present in the figure legend, table legend, main text, or Methods section.

n/a	Confirmed	
☐	☒	The exact sample size (*n*) for each experimental group/condition, given as a discrete number and unit of measurement
☐	☒	A statement on whether measurements were taken from distinct samples or whether the same sample was measured repeatedly
☐	☒	The statistical test(s) used AND whether they are one- or two-sided<br>*Only common tests should be described solely by name; describe more complex techniques in the Methods section.*
☒	☐	A description of all covariates tested
☒	☐	A description of any assumptions or corrections, such as tests of normality and adjustment for multiple comparisons
☐	☒	A full description of the statistical parameters including central tendency (e.g. means) or other basic estimates (e.g. regression coefficient) AND variation (e.g. standard deviation) or associated estimates of uncertainty (e.g. confidence intervals)
☐	☒	For null hypothesis testing, the test statistic (e.g. *F*, *t*, *r*) with confidence intervals, effect sizes, degrees of freedom and *P* value noted<br>*Give P values as exact values whenever suitable.*
☒	☐	For Bayesian analysis, information on the choice of priors and Markov chain Monte Carlo settings
☒	☐	For hierarchical and complex designs, identification of the appropriate level for tests and full reporting of outcomes
☒	☐	Estimates of effect sizes (e.g. Cohen's *d*, Pearson's *r*), indicating how they were calculated

Our web collection on statistics for biologists contains articles on many of the points above.

Software and code

Policy information about availability of computer code

Data collection	Leginon; SerialEM
Data analysis	CryoSPARC; Patch Motion; cryoSPARC Live; ModelAngelo; Coot; Rosetta; ISOLDE; Phenix; MolProbity; UCSF Chimera; UCSF ChimeraX; Prevateer; FlowJo; Prism; Inkscape; Biorender

For manuscripts utilizing custom algorithms or software that are central to the research but not yet described in published literature, software must be made available to editors and reviewers. We strongly encourage code deposition in a community repository (e.g. GitHub). See the Nature Portfolio guidelines for submitting code & software for further information.

Data

Policy information about availability of data

All manuscripts must include a data availability statement. This statement should provide the following information, where applicable:

- Accession codes, unique identifiers, or web links for publicly available datasets
- A description of any restrictions on data availability
- For clinical datasets or third party data, please ensure that the statement adheres to our policy

The final unsharpened map and the final sharpened map were deposited in the Electron Microscopy Data Bank under accession number EMD-48102, EMD-70264, EMD-48093, and EMD-48101 for NCS.1.1/CA09 sNAp [C4], NCS.1.1/CA09 sNAp [C1], NCS.1/DB16 NA (4 fabs), and NCS.1/DB16 NA (3 fabs), respectively. The final

structures was deposited in the PDB under accession numbers 9EJF, 9O9V, 9EIT, and 9EJE for NCS.1.1/CA09 sNAp [C4], NCS.1.1/CA09 sNAp [C1], NCS.1/DB16 NA (4 fabs), and NCS.1/DB16 NA (3 fabs), respectively.

Research involving human participants, their data, or biological material

Policy information about studies with human participants or human data. See also policy information about sex, gender (identity/presentation), and sexual orientation and race, ethnicity and racism.

Reporting on sex and gender	We report on sex.
Reporting on race, ethnicity, or other socially relevant groupings	Race and ethnicity are reported by participants.
Population characteristics	Participants are adults, 18 years and older, who are willing to donate blood or other samples that will be stored indefinitely and used for future research. Study participants have a condition that is of interest to research conducted in or supported by the Vaccine Research Center. Conditions of interest include infections of public health importance and receipt of vaccines to prevent them.
Recruitment	Recruitment is conducted with IRB-approved advertisements and using listserv email distributions, flyers, online media, and in-person community events to recruit study participants.
Ethics oversight	The NIH Institutional Review Board provides ethics oversight for the VRC 200 protocol.

Note that full information on the approval of the study protocol must also be provided in the manuscript.

Field-specific reporting

Please select the one below that is the best fit for your research. If you are not sure, read the appropriate sections before making your selection.

☒ Life sciences ☐ Behavioural & social sciences ☐ Ecological, evolutionary & environmental sciences

For a reference copy of the document with all sections, see nature.com/documents/nr-reporting-summary-flat.pdf

Life sciences study design

All studies must disclose on these points even when the disclosure is negative.

Sample size	Sample sizes of animal studies were determined based on prior experience with similar experiments. Assuming variance in the response is proportional to mean for a given group (constant CV of 30%, typical for this type of experiments), a group size of 10 will give 89% power to detect 2-fold differences or a 49% power to detect 1.5-fold differences between groups based on a two tailed test of means with an alpha set to 0.05 (calculation was performed by 1-way ANOVA pairwise tools at powerandsamplesize.com).
Data exclusions	No data were excluded.
Replication	Structural determination of NA-Fab complexes was performed once. All in vitro assays were repeated at least twice with similar results. Animal infection studies were performed once.
Randomization	All animals used in the study were randomly assigned to different experimental groups.
Blinding	All in vitro experiments were not performed blindly. All in vivo experiments were performed blindly to animal handlers.

Reporting for specific materials, systems and methods

We require information from authors about some types of materials, experimental systems and methods used in many studies. Here, indicate whether each material, system or method listed is relevant to your study. If you are not sure if a list item applies to your research, read the appropriate section before selecting a response.

Materials & experimental systems

n/a	Involved in the study
	☒ Antibodies
	☒ Eukaryotic cell lines
☒	Palaeontology and archaeology
	☒ Animals and other organisms
☒	Clinical data
☒	Dual use research of concern
☒	Plants

Methods

n/a	Involved in the study
☒	ChIP-seq
	☒ Flow cytometry
☒	MRI-based neuroimaging

Antibodies

Antibodies used: This study reports novel antibodies to influenza NA: NCS.1, NCS.1.1, NCS.1.2, NCS.1.3, NCS.1.4, and NCS.1.5. Other published antibodies (1G01, 1G05, 2E01, FNI9, DA03E17, VRC01) were used as controls.

Validation: All antibodies used in the study were confirmed to have desired sequences, specificities and activities prior to use in the study.

Eukaryotic cell lines

Policy information about cell lines and Sex and Gender in Research

Cell line source(s): MDCK-SIAT1-PB1 (Creanga et al. Nat Commun. 2021), Expi293 (ThermoFisher, A14527)

Authentication: Cell lines were not authenticated.

Mycoplasma contamination: Tested negative (monthly).

Commonly misidentified lines (See ICLAC register): n/a

Animals and other research organisms

Policy information about studies involving animals; ARRIVE guidelines recommended for reporting animal research, and Sex and Gender in Research

Laboratory animals: BALB/c mice

Wild animals: n/a

Reporting on sex: This study used only female mice because of the previous LD50 titration studies that were performed in female mice.

Field-collected samples: n/a

Ethics oversight: All experimental procedures conducted at University of Melbourne were approved by the University of Melbourne Animal Ethics Committee (protocol #22954). All animal procedures conducted at University of Pittsburgh were approved by the University of Pittsburgh Institutional Animal Care and Use Committee, IACUC (protocol #22040682), with animal care in accordance with the Guide for the Care and Use of Laboratory Animals (National Research Council) and the Association for Assessment and Accreditation of Laboratory Animal Care (AAALAC).

Note that full information on the approval of the study protocol must also be provided in the manuscript.

Plants

Seed stocks: *Report on the source of all seed stocks or other plant material used. If applicable, state the seed stock centre and catalogue number. If plant specimens were collected from the field, describe the collection location, date and sampling procedures.*

Novel plant genotypes: *Describe the methods by which all novel plant genotypes were produced. This includes those generated by transgenic approaches, gene editing, chemical/radiation-based mutagenesis and hybridization. For transgenic lines, describe the transformation method, the number of independent lines analyzed and the generation upon which experiments were performed. For gene-edited lines, describe the editor used, the endogenous sequence targeted for editing, the targeting guide RNA sequence (if applicable) and how the editor was applied.*

Authentication: *Describe any authentication procedures for each seed stock used or novel genotype generated. Describe any experiments used to assess the effect of a mutation and, where applicable, how potential secondary effects (e.g. second site T-DNA insertions, mosiacism, off-target gene editing) were examined.*

Flow Cytometry

Plots

Confirm that:

- [x] The axis labels state the marker and fluorochrome used (e.g. CD4-FITC).
- [x] The axis scales are clearly visible. Include numbers along axes only for bottom left plot of group (a 'group' is an analysis of identical markers).
- [x] All plots are contour plots with outliers or pseudocolor plots.
- [ ] A numerical value for number of cells or percentage (with statistics) is provided.

Methodology

Sample preparation	Cryo-preserved PBMCs were used.
Instrument	BD Symphony A5 (analyzer) and S6 (sorter)
Software	FloJo 10
Cell population abundance	This study reports a subset of cells from previous publication (Lederhofer et al. Immunity 2024).
Gating strategy	This study reports a subset of cells from previous publication (Lederhofer et al. Immunity 2024). Gating scheme was reported in the previous publication. Final gating of NA-specific B cells is shown in Supplementary fig. 1b.

- [x] Tick this box to confirm that a figure exemplifying the gating strategy is provided in the Supplementary Information.
